# CAP protein superfamily members in *Toxocara canis*

**DOI:** 10.1186/s13071-016-1642-y

**Published:** 2016-06-24

**Authors:** Andreas J. Stroehlein, Neil D. Young, Ross S. Hall, Pasi K. Korhonen, Andreas Hofmann, Paul W. Sternberg, Abdul Jabbar, Robin B. Gasser

**Affiliations:** Faculty of Veterinary and Agricultural Sciences, The University of Melbourne, Parkville, VIC Australia; Structural Chemistry Program, Eskitis Institute, Griffith University, Brisbane, Australia; HHMI and Division of Biology and Biological Engineering, California Institute of Technology, Pasadena, CA USA

**Keywords:** Nematodes, *Toxocara canis*, CAP (SCP/TAPS) protein genes, Transcription profiles, Genetic interactions

## Abstract

**Background:**

Proteins of the cysteine-rich secretory proteins, antigen 5 and pathogenesis-related 1 (CAP) superfamily are recognized or proposed to play roles in parasite development and reproduction, and in modulating host immune attack and infection processes. However, little is known about these proteins for most parasites.

**Results:**

In the present study, we explored CAP proteins of *Toxocara canis*, a socioeconomically important zoonotic roundworm. To do this, we mined and curated transcriptomic and genomic data, predicted and curated full-length protein sequences (*n* = 28), conducted analyses of these data and studied the transcription of respective genes in different developmental stages of *T. canis*. In addition, based on information available for *Caenorhabditis elegans*, we inferred that selected genes (including *lon-1, vap-1*, *vap-2*, *scl-1, scl-8* and *scl-11* orthologs) of *T. canis* and their interaction partners likely play central roles in this parasite’s development and/or reproduction via TGF-beta and/or insulin-like signaling pathways, or via host interactions.

**Conclusion:**

In conclusion, this study could provide a foundation to guide future studies of CAP proteins of *T. canis* and related parasites, and might assist in finding new interventions against diseases caused by these parasites.

**Electronic supplementary material:**

The online version of this article (doi:10.1186/s13071-016-1642-y) contains supplementary material, which is available to authorized users.

## Background

*Toxocara canis* (Werner, 1782) of canids is recognized as the principal causative agent of toxocariasis; this parasitic worm has a complex life cycle, which can also involve rodents and other animals as paratenic hosts [1]. In humans, particularly children, following the accidental ingestion of infective eggs of *T. canis*, infective larvae penetrate the intestinal wall and invade various tissues, leading to ocular larva migrans (OLM), visceral larva migrans (VLM), neurotoxocariasis (NT) and/or covert toxocariasis (CT) [2]. Some reports (e.g. [3, 4]) have also shown an association between *T. canis* infection or toxocariasis and allergic disorders in humans, such as urticaria, chronic pruritus and/or asthma. The *T. canis*-mouse and -dog models [5, 6] provide practical tools for studying *T. canis* biology, parasite-host interactions and human toxocariasis.

Some excretory/secretory (ES) proteins from *T. canis* likely play key roles in these interactions and the infection process, supported, to some extent, by recent research showing abundant transcription of genes encoding peptidases, cell-adhesion molecules (including integrins and cadherins) and lectins (C-type) in parasitic stages of *T. canis* [7]. Moreover, the cysteine-rich secretory proteins, antigen 5 and pathogenesis-related 1 (CAP) superfamily [8], defined based on sperm-coating protein (SCP)-like extracellular domains (InterProScan codes: IPR014044 and IPR001283), represents some ES molecules inferred from the genome of *T. canis* (cf. [7]). Although six CAP proteins encoded in the draft genome of *T. canis* had been described and were predicted to be ES molecules [7], preliminary curation work suggested the presence of a larger panel of related molecules in this worm (Stroehlein et al., unpublished). In spite of the major socioeconomic importance of toxocariasis globally and some promise for CAP proteins as drug or vaccine targets (e.g. [9–11]), detailed information on this group of proteins in ascaridoids has been lacking.

The completion of a draft genome and transcriptomes for *T. canis* [7] using an Illumina-based sequencing approach provides an opportunity to explore, in detail, these CAP proteins and other key protein groups of this nematode. Therefore, in the present study, using available data sets, we now define the complement of CAP proteins, establish their sequence features, explore the transcription profiles of their genes in selected life cycle stages and tissues, and predict their roles in *T. canis*. This work should guide structural and functional studies of CAP proteins of *T. canis*, and might assist in finding new interventions against toxocariasis.

## Methods

For the present study, we utilized data from the published draft genome and developmental transcriptomes of *T. canis* from Denmark (NCBI BioProject accession no. PRJNA248777; WormBase [7, 12]); the draft genome of this parasite is 317 Mb and is predicted to have 18,596 protein-coding genes. Amino acid (aa) sequences (inferred from coding gene regions and *de novo*-assembled transcripts; cf. [7]) were subjected to protein domain/family/profile searches using InterProScan v.5.15.54 utilizing default settings [13]. Sequences with homology to one or more domains present in known CAP proteins were extracted, and *de novo*-assembled transcripts mapped to genomic scaffolds using BLAT [14]. These BLAT-transcript alignments (PSL files) as well as RNA-sequence reads that mapped to the genome (BAM files; cf. [7]) and original gene predictions (Maker2 GFF file; cf. [7]) were then loaded into the Integrative Genomics Viewer (IGV; [15]). All of this gene prediction information was then integrated to verify and define individual genes and corresponding transcripts. Final open reading frames (ORFs) were inferred using the program EMBOSS getorf v.6.4.0.0 [16].

Quality-filtered, decontaminated paired-end RNA-sequence reads were mapped to all 19,004 transcript sequences of *T. canis* [7]. For all life cycle stages and tissues investigated herein, genewise transcripts per million (TPM) values were calculated using the program RSEM v.1.2.11 [17]. These TPM values were then log-transformed [log_e_(TPM + 1)] to display transcription profiles for CAP protein-encoding genes in a heat map utilizing the gplots package (heatmap.2 function) in R v.3.2.4 [18].

Differential transcription analysis was conducted employing the program edgeR v.3.12.1 [19], using expected count values from RSEM as input. Genes were included in the analysis if they had more than one read count per million (CPM) in at least three samples (= biological replicates; cf. [7]). Differential transcription of genes was computed using a general dispersion of 0.43, considering a false discovery rate (FDR) value of ≤ 0.01 to be statistically significant. Log_2_-fold change (FC) values were employed to infer an upregulation (log_2_-fold change: > 2) of transcription of genes based on pairwise comparisons of stages/sexes/tissues.

Genetic interactions were inferred based on network analysis described previously [20, 21]. Identities between aa sequences encoded in *T. canis* and those from other free-living and parasitic worms represented in ParaSite within WormBase (WBPS5) were established using BLASTp v.2.2.28+ [22]. Pairwise global aa sequence similarities were calculated using the program EMBOSS Needle v.6.4.0.0 [16].

## Results and discussion

### CAP proteins encoded in *T. canis* and their features

Homology-based profile/domain searches using InterProScan and manual sequence curation predicted 28 CAP protein sequences (cf. [8]), which mapped to 23 genomic scaffolds (Additional file 1: Table S1). Subsequent mapping of *de novo*-assembled transcripts to these genomic scaffolds revealed 23 full-length (with start and stop codons at the beginning and end of the coding sequence, respectively) and five partial transcripts (Additional file 1: Table S1). Of the 28 protein sequences encoded by these transcripts, 25 were single- and three were double-domain CAP proteins, respectively. The features of these 28 predicted proteins (including length, InterProScan domains and signal peptides) are summarized in Additional file 1: Table S1. Specifically, the single-domain proteins (72 to 546 aa in total length; average length: 225 aa) were usually shorter than the double-domain proteins (416 to 490 aa in total length; average length: 449 aa) and exhibited 10–87 % sequence similarity among each other, whereas double-domain sequences had 21–44 % sequence similarity among each other upon overall pairwise aa sequence comparison.

Based on InterProScan analysis, 22 of the 28 predicted proteins belonged to the “cysteine-rich secretory protein, allergen V5/Tpx-1-related subfamily” (PTHR10334), of which 16 and 5 had the “allergen V5/Tpx-1 family” (PR00837) and “venom-allergen 5” (PR00838) signatures, respectively (Additional file 1: Table S1). All 28 proteins had the InterProScan signature IPR014044 (CAP) and contained a PR-1-like (SSF55797) domain. Additionally, 20 sequences had an SCP (SM00198), 27 a pathogenesis-related (G3DSA:3.40.33.10) and 25 a CAP (PF00188) domain (Additional file 1: Table S1). In addition, seven single-domain and two double-domain proteins belonged to “allergen V5/Tpx-1 related, conserved site CRISP family 1” (PS01009). In contrast, only two single-domain proteins and none of the double-domain proteins were assigned to “allergen V5/Tpx-1 related, conserved site CRISP family 2” (PS01010). Moreover, 11 single-domain proteins had a signal peptide that was absent from the other 14 single-domain proteins and all three double-domain proteins (Additional file 1: Table S1).

Amino acid sequence comparisons of *T. canis* CAP proteins with those of free-living and parasitic worms showed that most (*n* = 21) *T. canis* CAP proteins shared the highest sequence identity (36–89 %) to those of *Ascaris suum* (*Asu*) or *A. lumbricoides* (*Al*), ascaridoids of pigs and humans, respectively. Close homologs of one to two *T. canis* CAP proteins (39–83 % aa identity) were identified in the equine ascaridoid *Parascaris equorum* (*Pe*)*,* the free-living nematode *Panagrellus redivivus* (*Pr*) and the entomopathogenic nematodes *Steinernema monticolum* (*Smo*) and *Steinernema scapterisci* (*Ss*) (Additional file 1: Table S2). Next, we explored transcription profiles for all 28 individual genes in different life stages/sexes/tissues.

### Transcription profiles

**Fig. 1:**
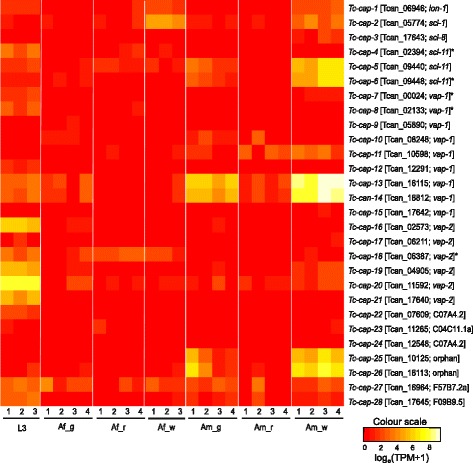
Heat map displaying transcription profiles for genes *Tc*-*cap-1* to *Tc*-*cap-28* in third-stage larvae (L3); adult female: gut (Af_g), reproductive tract (Af_r) and whole-worm (Af_w); adult male: gut (Am_g), reproductive tract (Am_r) and whole-worm (Am_w) of *Toxocara canis*; biological replicates are indicated (Lanes 1 to 3 or 1 to 4). The identifiers and closest *Caenorhabditis elegans* homologs of individual *Tc-cap* genes are in square brackets and partial sequences are indicated with an asterisk. The heat map was drawn based on log_e_-transformed “transcripts per million” (TPM) values (cf. Additional file 1: Table S4); a colour-scale indicates the level of transcription: low (*red*), medium (*orange*), high (*yellow*) and very high (*white*)

Subsets of the 28 genes encoding CAP proteins were differentially transcribed between/among different developmental stages, sexes and/or tissues (Fig. 1; Additional file 1: Table S3). Seven genes (*Tc-cap-4*, *Tc-cap-7*, *Tc-cap-16*, *Tc-cap-19*, *Tc-cap-20*, *Tc-cap-21* and *Tc-cap-22*) were consistently and highly upregulated in the third larval (L3) stage, with respect to all other developmental stages and tissues (FC-range: 2.3 to 15.8; Fig. 1; Additional file 1: Table S3). Two additional genes were significantly upregulated in L3 (FC-range: 3.5 to 6.2); one (*Tc-cap-18*) with respect to all adult male tissues and another (*Tc-cap-28*) in relation to all adult female tissues. Conversely, a significant upregulation (FC-range: 3.8 to 16.6) was seen in the adult male stage (whole-worm) of *T. canis* for *Tc-cap-5*, *Tc-cap-6*, *Tc-cap-13, Tc-cap-14, Tc-cap-25* and *Tc-cap-26* compared with all other stages and tissues. Thereof, four genes (*Tc-cap-13, Tc-cap-14, Tc-cap-25* and *Tc-cap-26*) were also highly transcribed in the gut and significantly upregulated compared with all other stages/tissues, except male whole-worm (Fig. 1), suggesting a role for these genes in digestion, but also in other processes, given the major upregulation in male whole-worm compared with male gut. Additionally, although not significantly upregulated with respect to the other two adult male tissues, *Tc-cap-11* was upregulated in the adult male whole-worm (FC-range: 4.8 to 8.0) compared with the female and L3 stages. Taken together, these findings suggest that these seven molecules play key roles in male reproduction, digestion and/or parasite-host interactions.

Although no significant female-enriched or -specific genes were inferred, *Tc-cap-2* was upregulated in both male and female whole-worms with respect to adult reproductive and gut tissues and the L3 stage, suggesting an exclusive role of this gene in both sexes of adult worms in tissues other than those studied, possibly by regulating longevity and/or stress resistance in mature worms, similar to the role that its ortholog (*scl-1*) plays in *Caenorhabditis elegans* [23].

The transcription profiles for *cap* genes of *T. canis* appear to be quite different from those of blood-feeding (haematophagous) nematodes, including the hookworms *Ancylostoma caninum*, *A. ceylanicum* and *Necator americanus*, in which *cap* genes are upregulated predominantly in parasitic larval stages [24–29], as well as the barber’s pole worm, *Haemonchus contortus*, where a substantial proportion of *cap* genes are highly transcribed in haematophagous stages [20]. There are also major differences in the numbers of CAP protein-encoding genes in *A. ceylanicum* (*n* = 432; [29]), *N. americanus* (*n* = 137; [30]) and *H. contortus* (*n* = 45; [20]) compared with *T. canis.* In addition, the relatively high proportion (93 %; *n* = 26; Fig. 1) of CAP proteins of *T. canis* with homologs in *C. elegans* contrasts the situation for *A. ceylanicum* and *N. americanus* [29, 30], for which only 14 of 432 (3 %) and six of 137 (4 %) CAP protein homologs, respectively, were identified in the free-living nematode. Taken together, these qualitative and quantitative differences indicate considerable variation among these nematodes in their biology and immune recognition, modulation or evasion mechanisms.

### Functional inferences

Of all 28 predicted CAP proteins of *T. canis,* 26 had *C. elegans* homologs (Additional file 1: Table S2). Specifically, all three double-domain proteins of *T. canis* had homologs in *C. elegans*, including *Ce*-VAP-1 (two homologs: *Tc*-CAP-7 and *Tc*-CAP-15), *Ce*-VAP-2 (one homolog: *Tc*-CAP-16) (Additional file 1: Table S2). Additionally, seven single-domain *Ce*-VAP-1 homologs and five single-domain *Ce*-VAP-2 homologs were detected (Additional file 1: Table S2). Of the remaining 13 single-domain proteins of *T. canis*, 11 had homologs in *C. elegans*, namely *Ce*-LON-1 (*Tc*-CAP-1), *Ce*-SCL-1 (*Tc*-CAP-2), *Ce*-SCL-8 (*Tc*-CAP-3), *Ce*-SCL-11 (*Tc*-CAP-4 to *Tc*-CAP-6), C07A4.2 (*Tc*-CAP-22 and *Tc*-CAP-24), C04C11.1a (*Tc*-CAP-23), F57B7.2 (*Tc*-CAP-27) and F09B9.5 (*Tc*-CAP-28) (Additional file 1: Table S2). For the two shortest full-length *T. canis* CAP protein sequences (*Tc*-CAP-25 and *Tc*-CAP-26; 90–95 aa), we did not detect any *C. elegans* homologs and thus labelled them “orphan” molecules (Fig. 1). However, both sequences did have homologs in a range of parasitic nematodes (see Additional file 1: Table S2), and *Tc*-CAP-26 also shared moderate aa sequence identity (46 %) with a sequence of the free-living nematode *Panagrellus redivivus*.

As most (26 of 28) *Tc*-CAP proteins predicted have homologs in *C. elegans* (Additional file 1: Table S2), we elected to infer functions from information available for the free-living worm in WormBase. We propose that the *C. elegans* LON-1 homolog, *Tc*-CAP-1, regulates body length and polyploidisation in hypodermal cells in adult and larval *T. canis*. In *C. elegans*, the protein encoded by *lon-1* is a target of TGF-beta signaling and is expressed in hypodermal tissues; the *lon-1* gene is negatively regulated by Sma/Mab pathway signaling and epistatic to *dbl-1* [31–33]. Knockdown of *lon-1* function in *C. elegans* by double-stranded RNA interference (dsRNAi) results in long worms, indicating that it is a negative regulator of body length [32]. *Ce-lon-1* encodes a protein (312 aa) with a sequence motif (GHYVQVVW) that is conserved in *T. canis Tc-cap-1* (284 aa) and across many other metazoan organisms (e.g. [32, 34–38]).

The *lon-1* gene interacts with *sma-2, -3* and *-4* (complex)*, -6, -9, -10, -12, -13, -14, -16, -17, -18* and *-19* encoding various Smad proteins (transcription factors), and *dbl-1* (see above)*, daf-4, kin-29* (encoding a kinase involved in regulating the expression of chemosensory receptors and entry into the dauer pathway)*, rnt-1, crm-1, lgg-1* and *che-2* (encoding a protein with G-protein beta-like WD-40 repeats that affects chemotaxis, longevity and dauer formation; expressed in male tail rays, and some head and sensory neurons [39–44]), most of which are involved (up- or downstream) in TGF-beta signaling. Given the relative conservation of *C. elegans* and *T. canis* LON-1 (overall aa similarity: 70 %), this protein might indirectly regulate body size, morphogenesis, germline quality and/or reproductive aging. In *C. elegans*, *lon-1* is expressed in the head region, intestine and hypodermis (cf. [12]; WBGene00003055); in *T. canis*, *Tc-lon-1* is transcribed at moderate levels in adult worms of both sexes, at low levels in the L3 stage and at very low levels in all other stages (Additional file 1: Table S4).

Both *Tc*-CAP-27 and *Tc*-CAP-28 are homologs of the *C. elegans* glioma pathogenesis-related protein 2 (GLIPR2) orthologs F57B7.2 and F09B9.5 (50–54 % aa identity), but these proteins share limited sequence similarity to human GLIPR2 (accession number: Q5VZR0; 16–30 % aa identity) and are slightly more similar to the human Golgi-associated plant pathogenesis-related protein 1 (GAPR1; PDB accession number: 4AIW; 18–34 % aa identity) of the same pathogenesis-related 1 (PR-1) family. Some of the genes encoding these proteins are well characterized in mammals [8, 38, 45]. In contrast to the moderate to low levels of transcription of F57B7.2 in the larval and young adult stages of *C. elegans* (cf. FPKM expression data from selected modENCODE libraries [46, 47]), *Tc-cap-27* and *Tc-cap-28* have variable transcription within and among the stages and tissues of *T. canis* studied here (Fig. 1), although *Tc-cap-27* is mostly transcribed at higher levels than *Tc-cap-28*. For *Tc-cap-27*, no significant upregulation was detected for any of the stages with respect to other stages/tissues, but *Tc-cap-28* was significantly upregulated in the L3 stage with respect to all tissues of female *T. canis* (FC-range: 5.2 to 6.2).

Originally, the *GLIPR2* gene was identified and localized to human chromosome 9p12-p13, with orthologous genes localized in syntenic regions in other species (cf. [8]). The GLIPR2 subfamily possesses several unique features that suggest that this subfamily might contain the most “primitive” mammalian CAP proteins. GLIPR2 proteins do not contain a predicted signal sequence, which is consistent with an intracellular localization to the Golgi membrane [48]. Interestingly, the conserved PY dipeptide sequence present in most other CAP superfamily members [8] is absent, as is the Hinge-like sequence present in other mammalian CAP proteins. In this latter respect, GLIPR2 is more like a non-mammalian CAP. This interpretation is supported by phylogenetic analysis [8], showing the GLIPR2 clade to be the “earliest diverged” CAP subfamily in mammals, using the protein PRY1 from the yeast *Saccharomyces cerevisiae* as the outgroup (cf. [49]). Analyses have shown *GLIPR2* orthologs to be present in a broad range of species including *S. cerevisiae*, *Drosophila melanogaster*, *C. elegans*, other nematodes, insects, ascidians, bony fish, amphibians, birds and mammals (e.g. [8, 20]). In mammals, experimental studies have shown that GLIPR2 is mainly expressed in/localized to tissues with immunological functions, including developing and circulating leukocytes within the spleen. High levels of GLIPR2 have also been localized in the embryo, kidney, lung, pancreas, placenta and uterus, and relatively low levels in the brain, skeletal muscles and testes [48, 50]. Taken together, these findings suggest that *Tc-cap-27* and *Tc-cap-28* might play an immunological role in the nematode and/or an immune modulatory role in the host animal.

The nine CAP proteins encoded by *Tc-cap-7* to *Tc-cap-15* are homologs of *Ce-*VAP-1 (Additional file 1: Table S2), which is a secreted protein similar to some venom allergen-like (VAL) proteins found in a number of invertebrates, including parasitic nematodes, and a human homolog of cysteine-rich secretory protein 3 isoform 2 precursor [51]. Transcription of the genes *Tc-cap-13* and *Tc-cap-14* was significantly upregulated in the adult male whole-worm (FC-range: 3.8 to 16.6) and male gut (FC-range: 3.7 to 12.7) with respect to all other stages/tissues of *T. canis* studied (Additional file 1: Table S3). Although the transcription levels of *Tc-cap-10* and *-11* were very low in the L3 and female stages, transcription in male stages was slightly higher than in females, whereas *Tc-cap-7*, *-8* and *-12* were transcribed at moderate levels in L3s, and *Tc-cap-9* and *Tc-cap-15* consistently at very low levels across all stages and tissues investigated (Additional file 1: Table S4). The set of nine VAP-1 homologs in *T. canis* suggests diversified functional roles of these molecules in this parasite, which appear to be reflected in considerable differences in transcription profiles. Based on functional data for *C. elegans*, we propose that at least some of the *Tc-cap* genes encoding VAP-1 homologs are involved in motility and expressed in chemosensory organs. In *C. elegans*, a *Ce-vap-1* reporter fusion is expressed specifically within sheath cells of such organs (i.e. amphids), and knockdown of *Ce-vap-1* results in an uncoordinated (Unc) phenotype (WBPhenotype:0000643; [52]). In addition, we propose that male-enriched expression of *Tc-cap-13* and *Tc-cap-14* in whole adult worms and gut tissues might relate to developmental and/or digestive processes, possibly acting in pathways associated with *Tc-cap-25* and *Tc-cap-26* (encoding “orphan” CAP proteins), which showed very similar transcription profiles.

Based on genetic network analysis, *Ce-vap-1* is inferred to interact with 17 *Ce-scl* genes (*Ce-scl-1*, *-2*, *-3*, *-5*, *-6*, *-7*, *-8*, *-9*, *-10*, *-11*, *-12*, *-13*, *-14*, -*15*, -*17*, -*18* and -*20*) in *C. elegans* [20, 53], and *Ce-scl-1* interacts with *age-1*, *daf-2* and *daf-16* [21, 23, 54–58]. Although *Ce*-*vap-1* and *Ce-vap-2* do not interact directly with each other, all 17 *Ce-scl* genes are predicted to interact with *Ce*-*vap-2* (cf. [20]) that interacts with *egl-9* [59]. Independent interactions of *Ce-vap-1* and *Ce-vap-2* with *Ce-scl-1*, and their association with parts of the insulin-like signaling pathway via *Ce-age-1*, *Ce-daf-2* and *Ce-daf-16* [20, 60] suggest an integrated but relatively complex role of these molecules/pathways in regulating nematode development and growth in *T. canis* and/or roles in the parasite-host relationship, which is supported by substantial differences in the number of *T. canis vap-1* (*n* = 9) and *scl* (*n* = 5) genes, compared with *C. elegans* (*n* = 1 and 26, respectively; [61]).

The *Ce-vap-2* homologs of *T. canis,* including *Tc-cap-16* to *Tc-cap-21*, represent homologs of human cysteine-rich secretory proteins involved in receptor-mediated endocytosis [62]. These receptors specifically recognize and bind extracellular macromolecules (ligands); the area of the plasma membrane with the receptor-ligand complex then undergoes endocytosis and forms a transport vesicle containing the receptor-ligand complex. Although involved in the specific uptake of particular substances (e.g. iron or low density lipoproteins) required by cells, endocytosis has also been linked to the transduction of signals from the periphery of cells to their nuclei [63]. The upregulation of *Tc-cap-16*, *-19*, *-20* and *-21* in the L3 stage might indicate a substantial requirement for nutrient uptake as the L3 stage enters the host animal and undergoes migration. Interestingly, *Tc-cap-18* is upregulated both in this stage and in the female adult reproductive tract (compared with all male tissues; FC-range: 3.5 to 6.2), which further supports the notion of a role for *vap-2* orthologs in nutrient uptake by both L3s and by progeny within gravid females. This proposal is relatively consistent with the finding that *Ce-vap-2* genetically interacts with *Ce-egl-9* (cf. [20]), a gene involved in yolk uptake by oocytes in *C. elegans* [64]. Functional genomic studies have shown that *Ce*-*vap-2* suppresses *egl-9*, leading to an egg-laying defect (Egl phenotype; WormBase entry WBRNAi00071630; [62]). The protein *Ce-*EGL-9 is known to function in a conserved hypoxia-sensing pathway to negatively regulate *Ce*-HIF-1 (hypoxia-inducible factor) by hydroxylating prolyl *Ce*-HIF-1 residues [65]; EGL-9 activity is negatively regulated by its physical association with *Ce*-CYSL-1, a protein with similarity to cysteine synthases that transduces signals linked to environmental O_2_ levels via hydrogen sulfide (H_2_S) signaling [66]. *Ce*-EGL-9 belongs to a protein superfamily (representing leprecan and AlkB) implicated in the hydroxylation of proteins and oxidative detoxification of alkylated bases [67]. This protein is expressed in hypodermis, musculature and neurons, and needed for muscle function for egg laying [68]. Based on this information, we suggest that *Tc-vap-2* is involved in aspects of reproduction, including egg-yolk uptake and egg laying, in intimate association with a *Ce*-*egl-9* ortholog and/or other complementary genes.

## Conclusions

Surprisingly, the CAP protein sequences of *T. canis* predicted in the recent *T. canis* genome project [7] that have been curated and described in detail in the present work were not described in previous molecular studies (reviewed by [69–71]). The number of transcripts encoding various CAP proteins in *T. canis* compares with those inferred from transcriptomic and genomic sequence data sets for *A. suum* and *Trichuris suis* [72, 73], but is substantially less than those for *A. caninum, A. ceylanicum*, *N. americanus* and *H. contortus* [20, 24, 29, 30, 74–76]. The reasons for this apparent difference are unclear, but might relate to variation in developmental and reproductive biology as well as varying modes of host invasion and immune modulation or evasion among nematode species (cf. [9, 10]). Moreover, we found no evidence of neutrophil inhibitory factor (NIF) homologs amongst the predicted CAP proteins of *T. canis*. NIFs can be relatively abundant in ES products from some parasitic worms, such as hookworms [77]. For instance, SCP-1, a NIF homolog of *A. caninum*, has been reported to bind host integrin CR3 (CD11b/CD18), leading to the inhibition of neutrophil function [78, 79]. The absence of a NIF homolog in *T. canis* suggests that other molecules might assume NIF-like roles in this parasite.

The generation of improved genomic and transcriptomic assemblies for parasitic helminths (cf. [80, 81]) and the availability of expanded datasets for *T. canis* [7], as well as the characterization of the gene silencing machinery in *T. canis* and *A. suum* [7, 72] and advances in functional genomics of these ascaridoids [82, 83] will likely create opportunities for investigations of CAP protein-encoding genes and their products in different developmental stages of these nematodes. The development of a medium- to high-throughput gene-silencing screening system for *T. canis*, based on initial evidence of effective knockdown of some genes [82], could accelerate our understanding of essential orphan CAP proteins and their genes (e.g. *Tc-cap-25* and *Tc-cap-26*), and should elucidate their involvement in biological and developmental pathways in this important parasite, and in parasite-host interactions. In conclusion, this study provides a basis to guide future studies of CAP proteins of *T. canis* and related ascaridoids, and might assist in finding new interventions against diseases caused by these nematodes.

## Abbreviations

BLAST, basic local alignment search tool; CAP, cysteine-rich secretory proteins, antigen 5 and pathogenesis-related 1; CRISP, cysteine-rich secretory protein; dsRNAi, double-stranded RNA interference; ES, excretory/secretory; FC, log_2_-fold change; FPKM, fragments per kilobase million; GAPR1, Golgi-associated plant pathogenesis-related protein 1; GLIPR2, glioma pathogenesis-related protein 2; SCP, sperm-coating protein; TPM, transcripts per million; VAL/VAP, venom allergen-like (protein)

## Additional file

Additional file 1: Table S1. CAP protein-encoding genes of *Toxocara canis*. For each predicted protein, the genomic scaffolds to which they map, including the length of the inferred amino acid sequences and InterProScan matches (including domains, motifs and signal peptides), are shown. **Table S2.** Pairwise comparisons of 28 predicted *Toxocara canis* CAP protein sequences with homologs in *Caenorhabditis elegans* and parasitic nematodes including *Ascaris lumbricoides* (*Al*), *Anisakis simplex* (*As*), *Ascaris suum (Asu), Brugia malayi (Bm), Caenorhabditis japonica (Cj), Caenorhabditis sinica (Cs), Dirofilaria immitis (Di), Dracunculus medinensis (Dm),* *Elaeophora elaphi (Ee), Enterobius vermicularis (Ev), Loa loa (Ll), Litomosoides sigmodontis (Ls),* *Oesophagostomum dentatum (Od), Onchocerca ochengi (Oo), Onchocerca volvulus (Ov), Parascaris equorum (Pe), Panagrellus redivivus (Pr), Steinernema carpocapsae (Sc), Steinernema feltiae (Sf), Steinernema glaseri (Sg), Syphacia muris (Sm), Steinernema monticolum (Smo), Steinernema scapterisci (Ss)* and *Thelazia callipaeda (Tc)*, with gene identification (ID) and identity (%) indicated in brackets. **Table S3.** *Toxocara canis cap* genes (*Tc-cap*) differentially transcribed (upregulated; log_2_-fold change: > 2) in individual life stages/sexes/tissues (pairwise comparisons). **Table S4.** Transcription of *Toxocara canis cap* genes (*Tc-cap*) in different life stages/sexes/tissues. Values are transcripts per million (TPM). (XLSX 44 kb)
